# Dr. George Monroe Proctor

**Published:** 1908-03

**Authors:** 


Dr. George Monroe Proctor, of Shalersville, O., died at his home in that village February 3, 1908, aged 69 years. He graduated at the University of Buffalo in 1867.

